# Frequency and Risk Factors for Spontaneous Pneumomediastinum in COVID-19 Patients

**DOI:** 10.3389/fmed.2021.662358

**Published:** 2021-04-09

**Authors:** Tania Guadalupe Rodriguez-Arciniega, Erick Sierra-Diaz, Jesus Armando Flores-Martinez, Maria Elena Alvizo-Perez, Irlanda Nataly Lopez-Leal, Ana Luisa Corona-Nakamura, Hermes Ernesto Castellanos-Garcia, Alejandro Bravo-Cuellar

**Affiliations:** ^1^Department of Internal Medicine, Western National Medical Center (IMSS), Guadalajara, Mexico; ^2^Department of Public Health, University of Guadalajara, Guadalajara, Mexico; ^3^Department of Urology, Western National Medical Center (IMSS), Guadalajara, Mexico; ^4^Department of Radiology, Western National Medical Center (IMSS), Guadalajara, Mexico; ^5^Department of Anesthesiology, Western National Medical Center (IMSS), Guadalajara, Mexico; ^6^Department of Endocrinology, Western National Medical Center (IMSS), Guadalajara, Mexico; ^7^Department of Infectious Diseases, Western National Medical Center (IMSS), Guadalajara, Mexico; ^8^Department of Immunology, Western National Biomedical Center (IMSS), Guadalajara, Mexico

**Keywords:** spontaneous pneumomediastinum, spontaneous pneumothorax, SARS-CoV-2, complications, risk factors, case-control study

## Abstract

**Background:** Spontaneous pneumomediastinum (SPM) is an uncommon condition in COVID-19 patients. No information about outcome or risk factors is available at the time. The aim of this research is to report on the frequency and risk factors of spontaneous pneumomediastinum in COVID-19 patients.

**Materials and Methods:** An unmatched case-control study was carried out in a tertiary health-care facility for patients with COVID-19. Electronic files were reviewed to identify patients with confirmed COVID-19 infection by RT-PCR. Univariate analysis was used to describe demographic data. Mean differences were calculated using the Mann-Whitney test. Frequency and odds ratios were calculated by standard operations.

**Results:** A total of 271 patients were included in the study. Nine patients showed spontaneous pneumomediastinum and four of them presented associated spontaneous pneumothorax. The most common risk factors associated with poor outcomes in COVID-19 patients were not considered as risk factors for spontaneous pneumomediastinum development.

**Conclusion:** Spontaneous pneumomediastinum is an uncommon clinical feature in COVID-19 patients. More research is necessary to formulate statements regarding prevalence, risk factors, and outcome.

## Introduction

Based on a report from the World Health Organization (WHO) from March 10, 2021, the number of confirmed COVID-19 cases worldwide were 117,332,262 and 2,605,356 deaths (1). Mexico is one of the counties with the highest number of cases. In the beginning of March, the number of confirmed cases was 2,130,477 with a total of 190,923 deaths (1). To date, the problem is of major concern for public health around the world.

Although a definitive diagnosis of COVID-19 infection is made by using a reverse transcription polymerase chain reaction (RT-PCR) assay (2), several authors reported on the role and utility of chest CT scan as a recommended tool for clinical practice for COVID-19 patients (3–5). In April 2020, the Dutch Radiological Society developed CO-RADS to assess the suspicion of pulmonary involvement of COVID-19. The scale is from 1 to 5, from very low to very high involvement (6). Nowadays, the CO-RADS scale is used widely in our medical facility as a complementary tool to measure the extent of compromised pulmonary parenchyma in COVID-19 patients.

Radiological features such as spontaneous pneumothorax have been described in 1% of COVID-19 patients (7, 8). Spontaneous pneumomediastinum (SPM), unrelated to assisted ventilation, is another radiological feature reported in literature as a frequent complication and is related to a poor outcome (9). In general terms, SPM has a reported frequency in non-COVID-19 patients of 1 in 25,000 and is more common in children (10). To date, there is no reliable data on the frequency of SPM in COVID-19 patients, however, some clinical cases are available in international literature (11–15).

Based on the previous information, the aim of this work is to report on the frequency of spontaneous pneumomediastinum and the related risk factors in a tertiary health-care facility that was converted for the caring of COVID-19 patients.

## Materials and Methods

An unmatched case-control study was carried out at the Western National Medical Center in a 2-month period (July and August 2020). This research was performed under permission from the ethics committee, Number 14 CEI 20190123/ COFEPRIS 17 CI 14 039 114. Electronic files from COVID-19 patients were reviewed (chest CT scans and laboratory results) that looked for clinical features related to spontaneous pneumomediastinum. Suspicious cases were reviewed by six physicians (including 2 radiologists) in order to validate the diagnosis. Selected files were reviewed in order to obtain anthropometric data, clinical features, inflammatory biomarkers, comorbidities, and outcomes. Inclusion criteria for cases and controls were complete electronic files (medical notes, laboratory results, and digital chest CT scan images) from both genders older than 18 years and COVID-19 diagnosis by RT-PCR. Selected patients were managed at the Western National Medical Center COVID-19 units by at least one of the authors of this report. A data analysis report used percentages, absolute frequencies, means, standard deviations, and a 95% confidence interval. Statistical significance was evaluated by means of the Mann-Whitney *U*-test (*p* < 0.05). The frequency and Odds Ratio (OR) were calculated using standard methods. All data were processed using Excel® (Microsoft, Redmond, WA, USA) Open Epi (Open-Source Epidemiologic Statistics for Public Health, Bill and Melinda Gates Foundation, Emory University, Atlanta, GA, USA) and EpiInfo version 7.2.4 (Centers for Disease Control and Prevention, Atlanta, GA, USA).

## Results

A total of 319 electronic files from COVID-19 patients confirmed by RT-PCR assay SARS-CoV-2 were reviewed. Those files without chest CT scans or complete laboratory reports were excluded (*n* = 48). The files included in the study were 271, including cases and controls. From the patient cohorts, 36.5% were women and 63.5% were males. Demographic data is shown in Table 1.

**Table 1:** Demographic data from SARS-CoV-2 patients.

**Variable**	**Female (*n* = 99) Mean (*SD*: 95%CI)**	**Male (*n* = 172) Mean (*SD*: 95%CI)**	P^*^
Age (years)	59.1 (15.9: 55.9–62.33)	59.5 (15.0: 57.23–61.76)	>0.05
Weight (Kg)	77.7 (19.5: 73.8–81.5)	85.0 (18.5: 82.21–87.78)	<0.01
Height (meters)	1.6 (0.07: 1.58–1.61)	1.7 (0.06: 1.69–1.70)	<0.01
BMI	30.0 (7.3: 28.54–31.45)	29.0 (5.7: 28.14–29.85)	>0.05
Hospitalized (days)	11 (6.1: 9.78–12.21)	12.9 (7.5: 11.73–14.06)	<0.05

* *Mann-Whitney U-test. BMI, Body Mass Index; Kg, kilograms; SD, standard deviation*.

The percentage of deceased patients from the entire cohort was 36.5 (*n* = 99/271), corresponding to 23.9% for males and 12.6% for females. Comorbidities from the total sample were obtained from files. Details related to such are shown in Table 2.

**Table 2:** Comorbidities measured in the general population with COVID-19 diagnosis (*n* = 271).

**Comorbidity**	**Female (*n* = 99) *n* (%)**	**Male (*n* = 172) *n* (%)**	***P* Fisher's exact test**
Tobacco smoking	9 (9.09)	28 (16.3)	>0.05
^*^COPD/Asthma	5 (5.05)	12 (6.9)	>0.05
Diabetes Mellitus	46 (46.4)	67 (39)	>0.05
Hypertension	62 (62.6)	97 (56.4)	>0.05
Chronic kidney disease	11 (11.1)	19 (11)	>0.05
Pneumothorax	0 (0)	4 (2.3)	>0.05
Pneumomediastinum	2 (2.02)	7 (4)	>0.05

* *Chronic obstructive pulmonary disease*.

**Figure 1:**
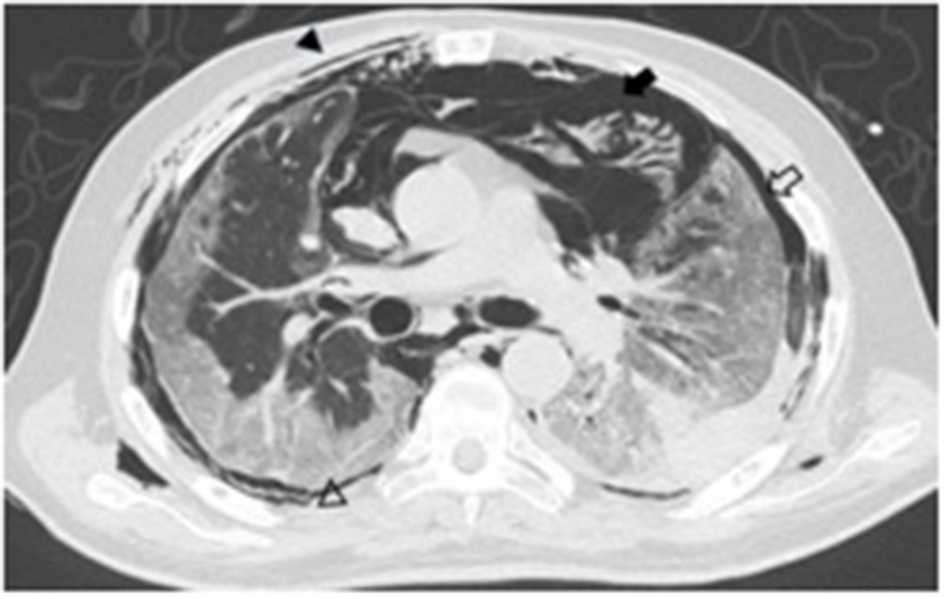
A 68-year-old male axial non-enhanced chest CT image showing subcutaneous emphysema (arrow heads), pneumomediastinum (black arrow), and pneumothorax (empty arrow).

**Figure 2:**
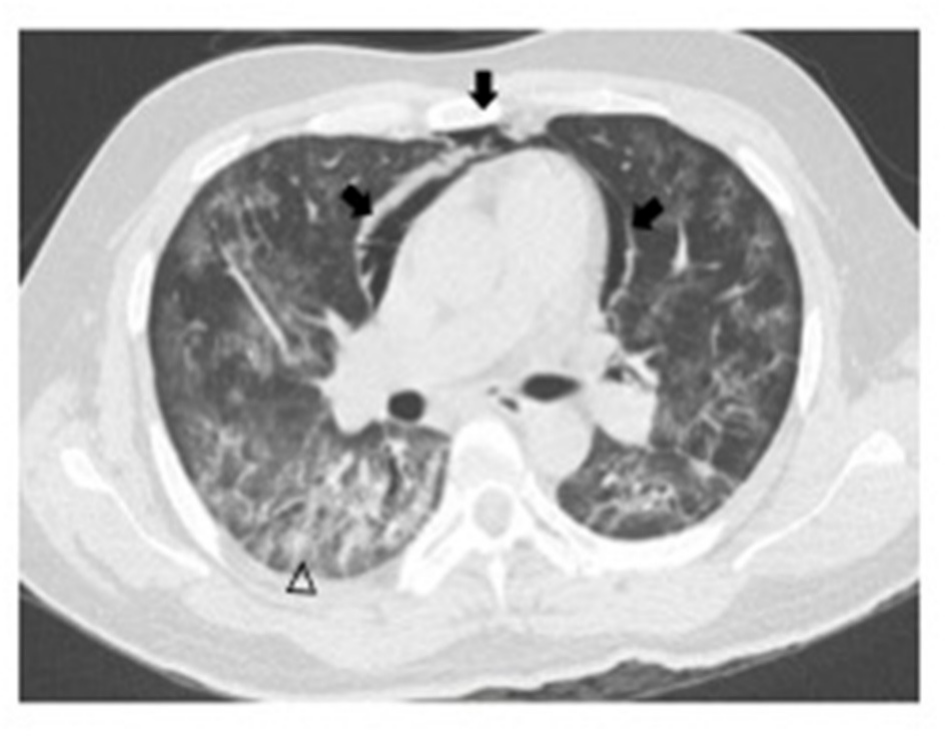
A 43-year-old male non-enhanced chest CT image showing pneumomediastinum (arrows) and ground glass opacity pattern over peripheral subpleural areas (arrowhead).

Based on the results, a total of 9 COVID-19 hospitalized patients developed spontaneous pneumomediastinum (3.3%), and 4 cases of spontaneous pneumothorax were detected (1.47%). All patients underwent a non-enhanced chest CT scan immediately after hospital admission, upon which SPM was identified by a COVID-19 physician team (Figures 1, 2). None of them received invasive mechanical ventilation at the time they were admitted. The principal characteristics of patients who developed SPM are described in Table 3. No significant differences were observed with regard to inflammatory biomarkers (*p* > 0.05). The main differences between non-spontaneous pneumomediastinum and SPM are shown in Tables 4, 5

**Table 3:** Features from COVID-19 patients who developed spontaneous pneumomediastinum.

**Variable**	**Cases (*n* = 9)** ** Mean (*SD*: 95%CI)**	**Controls (*n* = 262)** ** Mean (*SD*: 95%CI)**	P^*^
Age (years)	Mean 57 (15.26: 42.8–71.11)	59.5 (15.4: 58.02–61.77)	>0.05
Weight (Kg)	Mean 78.4 (17.8:64.71–92.08)	82.5 (19.3: 80.15–84.84)	>0.05
Height (meters)	Mean 1.67 (0.09: 1.60–1.73)	1.66 (0.09: 1.64–1.67)	>0.05
BMI	27.2 (5.2: 23.20–31.19)	28.8 (6.5: 28–29.59)	>0.05
Hospitalization (days)	16.8 (13.9: 6.11–27.48)	12.06 (6.7: 11.24–12.87)	>0.05

* *Mann-Whitney U-test. BMI, Body Mass Index; Kg, kilograms; SD, standard deviation*.

**Table 4:** Frequency of comorbidities in cases and controls.

**Comorbidity**	**Cases *n* (%)**	**Controls *n* (%)**
COPD^*^/Asthma	0	19 (7.3)
Diabetes mellitus	3 (33)	110 (42)
Hypertension	5 (56)	154 (59)
Tobacco smoking	1 (11)	36 (14)
Deaths	3 (33)	96 (36)

* *Chronic obstructive pulmonary disease*.

**Table 5:** Inflammatory biomarkers from cases and controls.

		**CRP mg/L**	**FER ng/mL**	**LDH U/L**	**LYMP × 10^**9**^/L**	**WBC × 10^**9**^/L**	**PLA × 10^**9**^/L**	**D-d ng/mL**	**PCT ng/mL**
Cases (*n* = 9)	Mean	119.8	1440.2	558.5	0.9	14.7	277.7	5441.1	0.5
	SD	88	852.7	272.5	0.3	8.3	107.2	7686.4	0.9
	95% CI	52–187	784–2,095	349–767	0.6–1.1	8.3–21	195–360	−467 to 11,349	−0.1 to 1.1
Controls (*n* = 262)	Mean	156.3	1084.6	484.3	0.9	11	266	2350.2	2.1
	SD	107.3	741.9	265.6	0.5	5.7	111.8	6780.9	8.6
	95%CI	143–169	994–1,174	451–516	0.8–0.9	10.3–11.6	252–279	1,525–3,175	1.0–3.1
p^*^		>0.05	>0.05	>0.05	>0.05	>0.05	>0.05	>0.05	>0.05

*CRP, C-reactive protein; FER, Ferritin; LDH, lactate dehydrogenase; LYMP, lymphocytes; WBC, white blood cells; PLA, platelets; D-d, D-dimer; PCT, procalcitonin*.

* *Mann-Whitney U-test*.

In order to calculate the Odds Ratio (OR), some independent variables (age and BMI) were adapted using a dichotomic approach that was used in the contingency tables. Age was considered as a risk factor if younger than 60-years old, and BMI was dichotomized in <25 and 25 or higher. Regarding gender, being male was considered as a risk factor. The ORs were calculated individually for each risk factor. Results obtained from the ORs are described in Table 6.

**Table 6:** Odds ratios obtained from contingency tables (case-control).

**Risk**	**Cases**	**Controls**	**OR (95%CI)**	***P* Fisher's Exact test**
Gender			2.05 (0.4–10.1)	>0.05
Male^*^	7	165		
Female	2	97		
Age (years)			1.2 (0.3–4.9)	>0.05
<60^*^	5	129		
≥60	4	133		
Tobacco smoking			0.7 (0.09–6.4)	>0.05
Positive	1	36		
Negative	8	226		
Diabetes mellitus			0.6 (0.16–2.8)	>0.05
Positive	3	110		
Negative	6	152		
Hypertension			0.8 (0.2–3.3)	>0.05
Positive	5	154		
Negative	4	108		
BMI			3.3 (0.85–12.7)	>0.05
<25^*^	4	51		
≥25	5	211		
Mortality			0.8 (0.2–3.5)	>0.05
Yes	3	96		
No	6	166		

The * means the risk factor for Spontaneous Pnemomediastinum. To be a male, younger than 60 years old and Body mass index < 25 are risk factors for spontaneous pneumomediastinum.

## Discussion

The results obtained in the present study clearly show that SPM is not always a pathologic feature related to invasive mechanical ventilation in COVID-19 patients. Indeed, main risk factors for SPM such as young age, tobacco smoking, asthma, and gender (10) did not show a significant statistical association. Other risk factors which are associated with poor outcomes in COVID-19 patients like hypertension and obesity were not statistically related to SPM either (*p* > 0.05). International research reported a frequency of 1 in 25,000 (10), however, our results showed an increased frequency of SPM in COVID-19 patients (9 in 271). The difference between proportions is remarkable (*p* < 0.01).

Spontaneous pneumomediastinum in COVID-19 patients has been reported as single cases in international literature (11–14). Other authors had reported some case series of SPM. Eperjesiova et al. reported 20 cases of air leak in a cohort of 976 COVID-19 patients. Five cases were SPM and were unrelated to mechanical ventilation. Two patients developed spontaneous pneumothorax, and the rest (*n* = 13) were air leak cases that were associated with medical procedures (15). However, the manuscript does not describe the relationship between SPM and risk factors.

During the peak of the SARS-CoV-2 pandemics in Spain, Gorospe et al. reported four cases of SPM that were unrelated to mechanical ventilation. None of the patients had a previous history of tobacco consumption or predisposing risk factors. One patient died after an infection of *Pseudomonas aeruginosa* (8). No statistical analysis of risk factors was performed for the study.

Recently, Jones et al. reported an observational study in England that included 83 critically ill COVID-19 patients. The authors divided the group into barotrauma and non-barotrauma groups. The barotrauma group included a total of 8 patients. One hundred percent of them had subcutaneous emphysema, seven revealed pneumomediastinum, and four had pneumothorax (bilateral *n* = 2). The seven patients that developed pneumomediastinum received ventilatory support through CPAP or non-invasive mechanical ventilation (NIV). The study reported significant mean differences between some variables, and a 9.6% of barotrauma as a complication in the cohort of patients. They assumed that barotrauma in COVID-19 patients is related to longer management prior to critical care admission and the use of CPAP or NIV. However, the manuscript does not specify if the chest CT scans were performed before or after CPAP management, NIV, and invasive mechanical ventilation (16).

The inflammatory process induced by the SARS-CoV-2 infection in the respiratory tract is characterized by an increase in intrathoracic pressure. Differential pressure inside the pulmonary parenchyma is the main cause of alveolar rupture that causes air leak through interstitial and bronchovascular tissues including the pneumomediastinum (17). The abnormal increase in pressure in the mediastinum causes air to dissect in between the mediastinal structures. The effect of dissection, which is secondary to air leak, extends from the soft tissue structure in the anterior mediastinum up to the subcutaneous tissue over the upper abdomen and neck (10). Symptoms are characterized by retrosternal chest pain, neck pain and swelling, dyspnea, dysphagia, and facial swelling (18).

In general terms, our results reveal that SPM is an uncommon condition resulting from an inflammation of the respiratory tract in COVID-19 patients. No specific data on the incidence of SPM is available at the moment, however, based on the local number of cases, the frequency was calculated in 3.3% of hospitalized COVID-19 patients. It is important to mention that the development of SPM does not increase the risk of death, nor the length of hospital stay.

From the total number of cases of SPM (*n* = 9), two of them showed a bilateral pneumothorax. One of these patients required invasive mechanical ventilation and died 3 days later. The other patient recovered successfully. Another two patients with SPM required invasive mechanical ventilation after diagnosis and died 3 and 5 days later, respectively. The other 6 patients recovered without mechanical invasive ventilation and were discharged around 16 days after admission.

## Conclusion

Development of SPM in COVID-19 patients is becoming an interesting topic nowadays. Information and data from international literature is still not enough to draw conclusions about specific causes, pathology, and outcome. More research and epidemiological data are necessary to make statements regarding this uncommon condition in patients affected by the novel SARS-CoV-2 virus.

## Data Availability Statement

The raw data supporting the conclusions of this article will be made available by the authors, without undue reservation.

## Ethics Statement

The studies involving human participants were reviewed and approved by this research was performed under permission from the ethics committee, Number 14 CEI 20190123/ COFEPRIS 17 CI 14 039 114. Instituto Mexicano del Seguro Social (IMSS). Written informed consent for participation was not required for this study in accordance with the national legislation and the institutional requirements.

## Author Contributions

All authors: participated directly in the care of COVID-19 patients. TR-A and ES-D: conceptualization, methodology, and writing-original draft preparation. ES-D: data analysis. TR-A, ES-D, JF-M, MA-P, IL-L, AC-N, and HC-G: investigation, formal analysis, validation, and sampling. TR-A, ES-D, JF-M, MA-P, IL-L, and HC-G: chest CT scan analysis. AC-N, TR-A, HC-G, and AB-C: writing-review and editing. AC-N, AB-C, and ES-D: project administration. All authors: have read and agreed to the published version of the manuscript.

## Conflict of Interest

The authors declare that the research was conducted in the absence of any commercial or financial relationships that could be construed as a potential conflict of interest.
